# Editorial: Assessment and management in violence and aggression

**DOI:** 10.3389/fpsyt.2024.1519741

**Published:** 2024-12-02

**Authors:** Howard Ryland, Stephanie Penney, Alexander Ian Frederic Simpson, Daniel Whiting

**Affiliations:** ^1^ Department of Psychiatry, University of Oxford, Oxford, United Kingdom; ^2^ Oxford Health NHS Foundation Trust, Oxford, United Kingdom; ^3^ The Centre for Addiction and Mental Health, Toronto, ON, Canada; ^4^ University of Toronto, Toronto, ON, Canada; ^5^ Institute of Mental Health, University of Nottingham, Nottingham, United Kingdom; ^6^ Nottinghamshire Healthcare NHS Foundation Trust, Nottingham, United Kingdom

**Keywords:** aggression, violence, forensic, mental health, risk, prediction

## Introduction

Absolute rates of violence in mental disorders are low and people with mental illness are similarly likely to be victims as perpetrators of violence (1–3). Violence associated with mental illness is however clinically relevant, as the risk is raised for people with certain mental illnesses relative to the general population (4–6). Violence is an important outcome both for individuals and from a public health perspective (7). Human costs include physical and psychological harms to victims and their families, and negative health, social and criminal justice consequences for perpetrators, such as those resulting from having a criminal conviction (8, 9). Direct economic costs to health systems result from activities such as mandatory or more intensive treatment, with indirect costs accruing to other systems, such as the criminal justice apparatus and across wider society, for example due to lost productivity (10).

While there is evidence that treating mental illness can reduce the risk of violence, it is a complex relationship with multiple causations (11–14). This necessitates a holistic approach to assessment and management that considers individual symptoms, behaviours, interpersonal relationships and social context (15). A better understanding of causal pathways to violence in those with mental illness would enable preventive measures to be developed and appropriately targeted (16). Risk assessment tools could allow insights from epidemiological studies to be operationalised to assist professionals in making more informed decisions across a range of clinical and other settings (17, 18). New strategies to reduce aggression are needed and must be robustly evaluated, such as through appropriately designed clinical trials (19).

## New research

This Research Topic brings together five papers examining many of these elements. It spans from investigations of how early childhood experiences relate to adult aggression, to an examination of the characteristics of people receiving mandatory treatment in China.

Recognising that the origin of aggression may lie in our early experiences, Koolschijn et al. studied the effect of childhood maltreatment on several outcomes, including aggression, in a sample of 128 forensic psychiatric patients. These authors found that higher scores on measures of childhood maltreatment were associated with higher aggression and violence risk assessment scores. They highlight the need to consider a patient’s history of maltreatment to guide risk assessment and treatment approaches. Notably, the presence and severity of childhood maltreatment is found to be a risk factor for violence and offending in the general population, as well as in clinical samples. This association has public health implications for prevention and early intervention, as initiatives to combat child maltreatment could be effective at reducing levels of aggression at a population level (20–22).

Schizophrenia is a condition with higher rates of violence compared to the general population, as well as to other forms of mental disorder (4). Sagayadevan et al. explored how schizophrenia symptom severity could mediate the relationship between aggression, impulsivity and quality of life outcomes in a sample of 397 mental health outpatients with schizophrenia-spectrum disorders in Singapore. Their analysis found indirect associations between motor impulsivity and self-control with various aspects of quality of life, through symptom severity. This helps elucidate one possible pathway by which impulsivity may impact on quality of life in this population.

A key step in reducing violent outcomes for clinical populations is to more readily and consistently integrate knowledge of predictors of violence into clinical practice to better individualise treatment (23–25). To do this effectively we need to have robustly validated risk prediction models tailored to the populations they are used in (26). Roaldset et al. validated a new violence risk screening tool for young people called V-RISK-Y, adapted from the well-established V-RISK-10 for adults (27). They found V-RISK-Y had an Area Under the Curve of 0.762 for violent behaviour in 67 adolescents admitted to a Norwegian emergency department. V-RISK-Y was also liked by staff who used it. The authors suggest changes to V-RISK-Y and propose further research to evaluate the revised tool.

An important issue relating to violence in psychiatric inpatient settings is the associated use of restrictive interventions (28). Whilst sometimes essential for safety, the practice raises ethical issues and there is increasing emphasis on minimising its use. Hirsch et al. investigated whether implementing new guidelines reduced coercion in German psychiatric wards. The paper presented here as part of the PreVCo randomised controlled trial (29), examines the baseline characteristics of 55 wards randomly allocated in matched pairs to the new guidelines or waiting list control. Coercion rates varied widely between wards, with an association between the frequency of coercive measures used and the percentage of involuntarily admitted cases. There was no difference in the rates of coercion between wards assigned to the intervention versus controls. Fidelity of guideline implementation also varied considerably among intervention wards. The paucity of randomised evidence of this nature in forensic settings is notable, so this study is significant for overcoming the many practical and ethical barriers to such work (30).

Concerns regarding imminent risk of violence is associated with mandatory treatment under many international mental health legal frameworks (31). Qiu et al. studied the characteristics of people subject to mandatory treatment in China under the Criminal Procedures Law of 2013. They found a year-on-year increase in the number of people subject to this provision from 2013, when the law was instituted, to 2019, followed by a sharp decline in 2020 and 2021, coinciding with the Covid-19 pandemic and associated restrictions. Most applications for mandatory treatment were approved and schizophrenia was the commonest diagnosis for people subject to mandatory treatment.

## Conclusion

This Research Topic highlights several key research priorities in violence risk assessment (32). It is encouraging to see systematic attempts to understand the origins and patterns of violence in people with serious mental illness, as this can advance efforts to provide more tailored interventions for different types of violence and symptoms. Risk assessment tools require careful refinement for utility and efficiency, to ensure they are operating in expected ways within the specific population of interest. As compared to other fields of medicine, evidence from randomised controlled trials is lacking, and the efforts of Hirsch et al. are especially notable in this regard. Finally, systems-level approaches, such as the one adopted by Qui et al., are under-utilised, but vital to place patient- and ward-level findings on the association between mental illness and violence in their appropriate context.
